# Potential of Waste Marble Sludge for Repressing Alkali-Silica Reaction in Concrete with Reactive Aggregates

**DOI:** 10.3390/ma15113962

**Published:** 2022-06-02

**Authors:** Ali Ahmed, Safeer Abbas, Wasim Abbass, Ayesha Waheed, Afia Razzaq, Elimam Ali, Ahmed Farouk Deifalla

**Affiliations:** 1 Department of Civil Engineering, University of Engineering and Technology, Lahore 54890, Pakistan; ali@uet.edu.pk (A.A.); safeer.abbas@uet.edu.pk (S.A.); ayeshawaheed3@gmail.com (A.W.); 2 Department of Architectural Engineering & Design, University of Engineering and Technology, Lahore 39161, Pakistan; afiarazzaq@uet.edu.pk; 3 Department of Civil Engineering, College of Engineering in AlKharj, Prince Sattam bin Abdulaziz University, AlKharj 16273, Saudi Arabia; i.ali@psau.edu.sa; 4 Civil Engineering Department, College of Engineering, Mansoura University, Mansoura 35516, Egypt; 5 Department of Structural Engineering, Faculty of Engineering and Technology, Future University in Egypt, New Cairo 11845, Egypt; ahmed.deifalla@fue.edu.eg

**Keywords:** alkali silica reaction, concrete, durability, expansion, reactive aggregates, waste marble sludge

## Abstract

The continuous development of the marble industry has led to an increase in the accumulation of waste marble sludge causing landfilling and health-associated issues. The intention of the current study is to explore the potential of waste marble sludge powder (MS) utilization as a means of controlling alkali-silica reaction (ASR) in concrete. Specimen (cubes, prisms, and mortar bars) were prepared to incorporate reactive aggregates and various proportions of MS ranging from 5% to 40% as a replacement for aggregates. Expansion and mechanical strength characteristics were determined to investigate the effectiveness of MS to control ASRfor up to 150 days. Results revealed that on replacing aggregates in the control specimen with 25% MS, the ASR expansion at 14 days reduced from 0.23% to 0.17%, and the expansion at 28 days reduced from 0.28% to 0.17% which is within limits as per American Standard for Testing of Materials (ASTM) C1260. Furthermore, specimens incorporating MS exhibited improved compressive and flexural strength as compared to the identical specimen without MS. Microstructural analysis using Scanning electron microscopy (SEM) revealed micro-cracks in the control specimen while the specimen incorporating MS was found intact. Thus, it can be foreseen that the use of MS as a partial replacement of aggregates can control ASR in concrete as well as reduce the dumping and harmful emissions issue.

## 1. Introduction

Alkali-Silica reaction (ASR) has arisento be one of the major concerns for civil engineers in recent decades [1,2,3]. ASR is a harmful reaction that occurs between the alkali hydroxide in pore solution and amorphous silica present in the reactive aggregates. The byproduct of this reaction is a deleterious gel (ASR-Gel). The extent ofgel production (an indirect measure of induced damage level) depends on various parameters including nature of alkalis [4,5,6], relative content amorphous of silica [7,8,9], humidity [10,11,12], temperature [5,11,13,14,15], aggregate size [7,16,17] and concrete stiffness [18,19,20,21]. This gel is hygroscopic in nature and expands in volume after absorbing water [22,23,24]. The increase in volume may cause cracks in surrounding concrete which may either cause instant damage or shorten the service life by reducing the durability of affected structures [23]. Various important structures have been reported to be damaged by ASR including MactaquacDam in Canada, Warsak and Terbela dams in Pakistan, Elgeseter bridge in Norway, and Paulo Guerra bridge in Brazil [25,26,27,28]. Early investigations on the effect of ASR on various properties of concrete report around 39% to 63% loss in compressive strength in case of amorphous and opal-based aggregates respectively, after 1 year of age. Furthermore, approximately 13% loss in flexural strength was also reported after only 28 days of casting concrete samples [29]. Similarly, substantial loss in the mechanical properties (compressive and flexural strength and modulus of elasticity) was also reported [30]. A research study carried out on the effect of ASR on reinforced concrete thick slabs reported excessive expansion in all directions [31].

Concerning the damage that might be induced by ASR gel, preventive measures of ASR should be taken by limiting at least one of the three main requisites of alkali-silica reaction i.e., reactive silica, alkali content, and high water content [1]. Therefore, supplementary materials may be used to replace either cement or reactive aggregates to control ASR in concrete by limiting the alkali content or the presence of reactive silica, respectively. The most widely reported remedial method targets the alkali content contributed by cement. Cement is replaced with supplementary cementitious materials to limit the alkali content which may react with reactive silica present in aggregates.Replacing cement with such supplementary materials reduces the alkali content (present in cement) which in turn reduces the extent of ASR in concrete [32,33,34,35].

One of the widely used natural products is marble which is used for producing various ornamental products as well as flooring and various other elements in structures. The production of marble products creates a lot of waste marble either in form of large pieces during the initial cutting stages or fine-grained waste marble sludge produced during the shaping or polishing of marble products [36,37,38,39]. It is reported that approximately 20–25% of the marble is wasted as either large pieces or fine-grained sludge during various production processes [40]. This waste is usually deposited in open landfills. Such landfills may create health risks for the workers working in these areas. The health risks associated with waste marble sludge are silicosis, lung cancer, and even cardiac issues [41,42,43].

One feasible method to safely dump this marble sludge waste is to utilize it in concrete. The use of supplementary materials in concrete has been vastly studied and reported. Various studies have been carried out on the performance evaluation of concrete incorporating different supplementary materials. Various studies have reported the improvement in the fresh state and hardened state mechanical properties of concrete on incorporating waste marble sludge powder (MS). Some of the most widely reported supplementary materials include fly ash, silica fumes, and powdered blast furnace slag. It has been reported that by incorporating such materials the mechanical characteristics of concrete may be improved [44,45,46,47,48]. For example, it has been reported that incorporating 5–10% of marble sludge in concrete may reduce the need for water for maintaining workability [49]. A similar trend of improvement in workability on the addition of MS in concrete was reported by several research reports [50,51,52]. The reduction in water demand may help in achieving better workability at the same water to cement ratio for various mixtures. Furthermore, it was reported that withthe incorporation of 15% and 20% MS in concrete, the porosity is reduced significantly [39,49]. Several research studies were also conducted on the effect of the incorporation of MS on the mechanical properties of concrete. The effect of replacing cement with various dosages of MS has been reported in earlier studies. The results present a plateau effect with the maximum gain in compressive strength corresponding to 20% cement replacement with MS. The trend reported corresponding to the flexural strength was similar to that of compressive strength results. The maximum increase in the flexural strength was reported corresponding to 20% cement replacement with MS [39]. Several other studies carried out on the utilization of MS or fine marble dust in concrete have reported improvement or nearly stable/maintained mechanical properties of concrete [49,50,51,52].

As ASR in concrete may be controlled by limiting the reactive silica by replacing reactive aggregates with various waste materials, this study explores the potential use of locally available MS for enhancing the concrete performance by controlling the expansion due to ASR. It is believed that by controlling ASR in concrete its durability may be increased as well as various mechanical properties may be improved. Therefore, reactive aggregates were replaced by MS in various proportions, and the impact on expansion due to ASR was studied along with a study of various mechanical properties.

## 2. Material and Mortar Mix Proportions

The concrete mixtures produced for investigating the impact of MS on ASR expansion, ordinary Portland cement (OPC), and reactive aggregates were utilized. The aggregates being produced at the quarries located in the Sargodha region were procured. MS was procured from a local marble tile production plant located in the Lahore region. Afterward, the sludge was then ground in a ball mill to achieve two fractions of particle sizes (i.e., smaller than 149µm and 75 µm). Aggregates were replaced in this study with 5%, 10%, 15%, 20%, 25%, 30% and 40% with MS (by weight). ASTM C1260 [53] was followed for producing various mixtures. As per the code, the cement to aggregate ratio is kept at 1:2.25 and the water to cement ratio was kept at 0.47. The aggregates are crushed to achieve the required gradation. According to ASTM the regular aggregates (to be used in concrete) should be crushed and mixed so that 10% should be passing the 4.75 mm sieve, 25% should be passing through each of the 2.36 mm, 1.18 mm, and 0.60 mm sieves and 15% passing 0.30 mm sieve. Table 1 shows the various proportions of MS used.

## 3. Experimental Procedures

Chemical analysis, scanning electron microscopy (SEM) and X-ray diffraction analysis (XRD) were conducted to determine the composition of cement, aggregates, and MS used in this study. Furthermore, the specific gravity and fineness were also evaluated following ASTM C188 [54] and ASTM C184 [55], respectively. The physical properties of aggregates (void content, bulk density & specific gravity, water absorption, impact value, crushing value, and abrasion value) and mechanical properties of aggregates were also determined following respective ASTM or BS guidelines. In addition, petrographic analysis of used aggregates was conducted following ASTM C295 [56]. Moreover, SEM analysis was carried out to investigate the properties of mortar specimens. To determine the pozzolanic potential of MS various tests were performed including differential thermal analysis (DTA) and thermo-gravimetric analysis (TGA). TGA and DTA analysis was carried out up to 1100 °C for specimens without MS and mixtures incorporating various dosages of MS after 28 days of curing.

**Figure 1:**
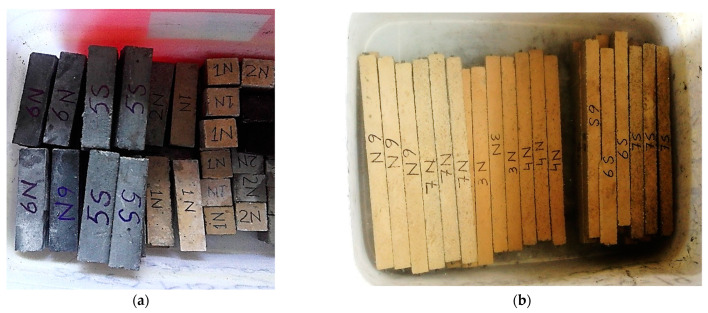
Specimens prepared (**a**) mortar cubes (50 × 50 × 50 mm) and prisms (40 × 40 × 160 mm) (**b**) mortar bars (25 × 25 × 285 mm).

Mortar cube and prism specimens of 50 × 50 × 50 mm and 40 × 40 × 160 mm were produced for compression strength and flexural strength tests (carried out using UTM), respectively. Five identical specimens were prepared for the test at each age regarding each mix proportion. Three identical mortar bars of size 25 × 25 × 285 mm were prepared for the test at each age, to determine ASR potential in accordance with ASTM C1260 [53] using three gang prism molds (Figure 1). A digital length comparator was used to determine the length of mortar bars at different ages. These mortar specimens were placed in 1N NaOH solution at 80 °C and tested at various ages (i.e., 7, 14, 21, 28 days). The microstructural and chemical composition of the selected specimen was also determined through scanning electron microscopy (SEM).

## 4. Results and Discussion

### 4.1. Raw Material Properties

**Figure 2:**
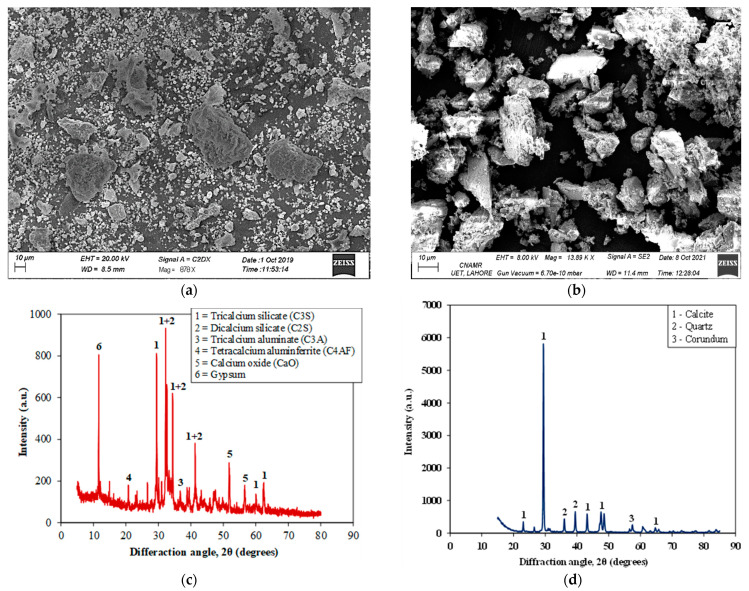
SEM and XRD patterns of raw materials (**a**) SEM image of cement (**b**) SEM image of MS (**c**) XRD pattern of cement (**d**) XRD pattern of MS.

The chemical properties of cement and MS used in this study have been presented in Table 2. It was observed that the chemical properties of the used cement were within the limits provided by ASTM C114 [57] (Table 2) except for the alumina content (Al_2_O_3_) which was found to be slightly below the lower limit. The equivalent alkali content present in the cement was below 0.6% which makes this cement well suited for expansion-related studies (ASTM C1260). The chemical properties of MS show lower alkali content in MS (0.13%). Lesser alkali contents in additives ensure reduced ASR in concrete. Figure 2 presents the microimages of used cement and MS obtained via SEM analysis. The particles of both the materials (cement and MS) were of irregular shape and non-uniform size. However, the particle size variation in cement was more diverse than in MS. XRD analysis of raw material was also conducted. It was observed that a high content of calcium oxide (CaO) was present in MS which was also confirmed through XRD analysis (Figure 2). Small quantities of other compounds were also found including Silica, alumina, ferrous, and Magnesium Oxide. The results from the XRD analysis of OPC cement and MS were used in this study. XRD analysis shows the presence of tri-calcium silicate (C_3_S) and di-calcium silicate (C_2_S) via higher peaks. Moreover, the presence of tetra-calcium alumino ferrite, tri-calcium aluminate, calcium oxide, gypsum, and magnesium oxide were also confirmed. XRD of MS showed that calcite was the major component present in MS while quartz and corundum were present in minor quantities. Loss on ignition (LOI) for MS was found to be 43% while it was observed to be only 4% in the case of cement. This higher LOI value in the case of MS may be attributed to decarbonation and emission of carbon dioxide (CO_2_). Similar findings have also been reported previously [49].

Table 3 presents various physical properties of Cement and MS. The specific gravity of cement was found to be 3.15 while the unit weight of cement was 1400. While these values for MS were 2.64 and 1206 which were lesser than that of cement. Furthermore, the fineness of MS was relatively higher as compared to cement. Hence, MS can be used as a filler material to improve packing and reduce porosity as also observed by previous studies [49,50]. The autoclave expansion of cement was found to be 0.13% which is less than 0.2% hence it can be used to assess the ASR potential according to ASTM C1260 [53].

**Figure 3:**
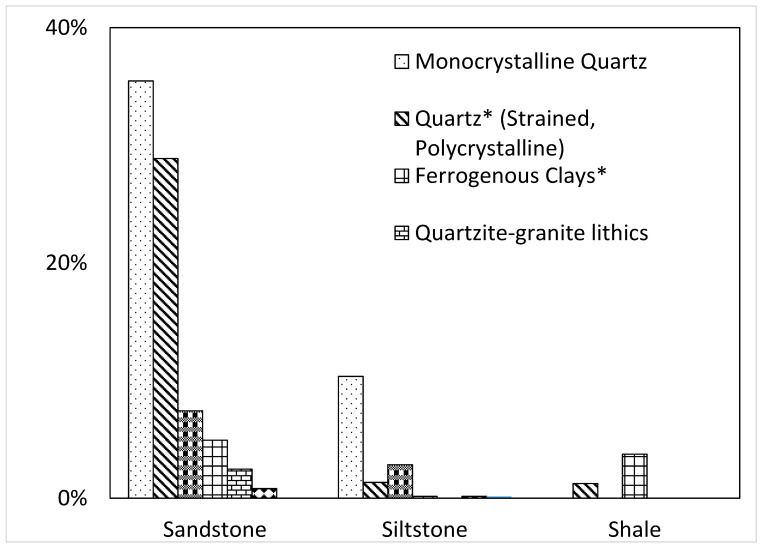
Petrographic examination of aggregates (* reactive components).

Results of physical and chemical examination of aggregates are presented in Table 4. It was observed that the sulfate content in the aggregates was quite negligible (0.043%). Silica was present in the highest quantity (56.92%). Other compounds found in aggregate were ferric oxide, calcium oxide, alumina, and magnesium oxide. The bulk density of aggregates was 1307.42 which was well within the range specified by ASTM C29 [58] (1200–1760). The specific gravity of aggregates was 2.6. The impact value and abrasion value of aggregates were 22.31% and 22.30% respectively. These were also observed to be below the upper limit of 23% for impact value and 50% for abrasion value as specified by ASTM C33 [59]. The petrographic analysis of the aggregates (Figure 3) showed that the aggregates were composed of shale, siltstone, and sandstone. Enough quantity of stained and polycrystalline quartz was found in the aggregates to confirm the possible reactive nature. Further, the presence of ferruginous clays indicated the high possibility of ASR reactiveness in the aggregates used. Detailed petrographic analyses have been presented elsewhere [47].

### 4.2. Effect of MS on Mechanical Performance of Mixtures

**Figure 4:**
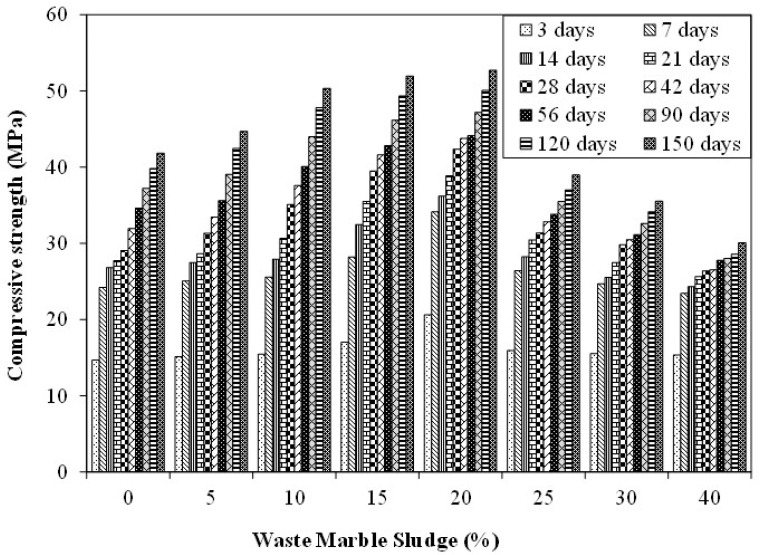
Compressive strength results.

Figure 4 shows the results of compressive strength tests for mixtures incorporating various proportions of MS at various ages (i.e., 3, to 150 days). These results are an average of five identical specimens of each type with a coefficient of variation lesser than 3%. Under normal conditions, the strength of the control specimen increased with age (as expected). The strength of the control specimen at 7, 28, 56, and 150 days was observed to be 24 MPa, 29 MPa, 35 MPa, and 42 MPa respectively. As compared to 7 days strength, the increase in strength after 28, 56, and 150 days was observed to be 20%, 43%, and 73% respectively. The strength of specimens incorporating MS also increased with age but at a relatively lower rate. The result shows that, withthe incorporation of MS, the compressive strength of the specimen improved in most of the cases. On incorporating 5% MS the strength achieved at 7, 28, 56 and 150 days was 25 MPa, 31 MPa, 36 MPa, and 45 MPa respectively. This accounts for a 25%, 42%, and 78.2% increase in compressive strength at 28, 56, and 150 days respectively. Similarly, the compressive strength achieved on incorporating 20% MS after 7, 28, 56, and 150 days was observed to be 34 MPa, 42 MPa, 44 MPa, and 53 MPa respectively. The corresponding percentage increase in strength was calculated to be 24%, 29%, and 55% respectively. The results show an increase in the compressive strength with an increase in curing age, which may be contributed to the continuous hydration process due to the presence of un-hydrated cement particles and water.

Moreover, the effect of the content of MS on the compressive strength of mortar was observed as a plateau effect. Specimens with 15% and 20% MS exhibited a maximum increase in the compressive strength at various ages. For instance, at 28 days the compressive strength of specimen incorporating 5%, 15%, 20%, and 40% MS was observed to be 31 MPa, 40 MPa, 42 MPa, and 26 MPa respectively. It may be observed that although the compressive strength with 20% MS was around 46% more than the control sample, the compressive strength of the specimen with 40% MS was reduced by around 9%. Similarly, at 150 days the compressive strength of specimen incorporating 5%, 15%, 20%, and 40% MS was observed to be 45 MPa, 52 MPa, 53 MPa, and 30 MPa respectively. This corresponds to an increase of 24% and 26% at 15% and 20% MS replacement while a reduction of 28% was observed in specimens with 40% MS replacement. Results show an increasing trend of up to 20% MS content. The compressive strength was observed to reduce in specimens with more than 20% MS. At higher ages, the specimen with 25% and more content of MS exhibited lesser strength as compared to the control specimen. Ahmed etal. reported that withthe addition of 10% and 20% marble sludge the compressive strength increased by 2.7% and 9.7% respectively [39]. The increase in compressive strength on the addition of MS may be attributed to two phenomena. First of these is the filling effect of MS which improves the packing of aggregates. This process would work even with inert filler materials that would fill in the pores [60,61]. Furthermore, this process may enhance the properties of the transition zone (TZ) surrounding the aggregates [62]. The secondphenomenon is related to the action of MS fines as nucleation sites for CH and C-S-H products. This cause an increase in strength at the early stages due to the hydration of clinker minerals [60,63,64]. Further, due to the potential hydration of calcite and C_3_A, the chemical binding occurred as well as the porosity of the resulting mixtures was reduced.

**Figure 5:**
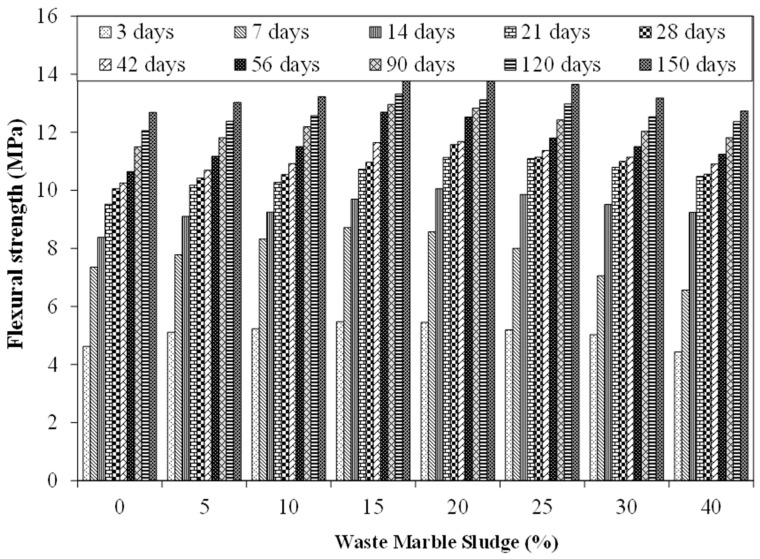
Flexural strength results.

Figure 5 shows the results of flexural strength tests for mixtures incorporating various proportions of MS at various ages (i.e., 7 to 150 days). These results are an average of five identical specimens of each type with a coefficient of variation lesser than 3%. The strength of the control specimen at the age of 7, 28, 56, and 150 days was observed to be 7.4 MPa, 10.0 MPa, 10.7 MPa, and 12.7 MPa respectively. The flexural strength of the specimen incorporating 5% MS was observed to be 7.8 MPa, 10.4 MPa, 11.2 MPa, and 13.0 MPa after 7, 28, 56, and 128 days. While the strength of the specimen incorporating 15% MS was observed to be 8.7 MPa, 11.0 MPa, 12.7 MPa, and 14.1 MPa at 7, 28, 56, and 150 days respectively. The flexural strength of the specimen showsa plateau effect with maximum strength observed in the case of specimens with 15% MS content. The flexural strength of specimens containing more than 20% MS starts reducing. For instance, the flexural strength of the specimen at 28 days was observed to be 10.4 MPa, 11 MPa, and 10.6 MPa at 5%, 15%, and 40% MS content. Similarly, the flexural strength of the specimen at 150 days was observed to be 13.0 MPa, 14.0 MPa, and 12.7 MPa at 5%, 15%, and 40% MS content. Hence, it can be concluded from these results that 15% incorporation of MS gives the highest flexural strength under normal conditions. As the flexural strength test results show a trend similar to compressive strength test results, it can be concluded that the increase in flexural strength could be attributed to the same reasons as discussed earlier in the case of compression test results. Out of the filler effect of MS and possible chemical reaction developed between hydration of calcite and C_3_A, the latter might be considered the main contributor in the case of flexural strength improvement.

### 4.3. Expansion Due to ASR

**Figure 6:**
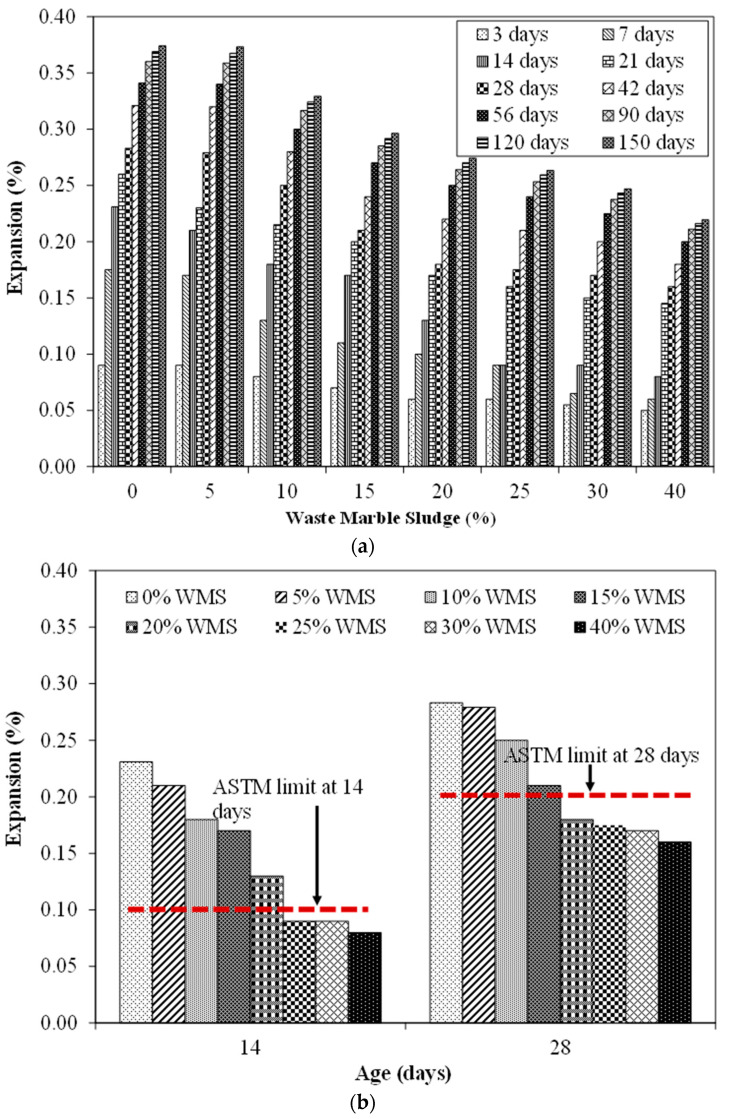
Expansion results (**a**) at all ages (**b**) at 14 days and 28 days with ASTM limits.

The expansion results of mortar bars having different proportions of the unprocessed MS placed in NaOH solution at 80 °C are shown in Figure 6. The results presented are an average of three identical specimens with a coefficient of variation of less than 2%. Results revealed that the control specimen exhibited an expansion of 0.23% at 14 days and 0.28% at 28 days. According to ASTM C1260, the expansion of specimens at 14 days should be lesser than 0.1% while at 28 days, it should be lesser than 0.2%. Therefore, the control specimens may be considered alkali reactive as per ASTM C1260. Moreover, the expansion exhibited by the control specimen was more than the limits prescribed by RILME AAR-2 [65] and Australian Standard AS-1141.60.1 [66]. RILEM AAR-2 recommends a maximum expansion of 0.1% at 16 days while Australian Standard AS-1141.60.1 recommends a maximum expansion of 0.10% at 10 days and 0.3% at 21 days. The specimens exhibited an increase in expansion with an increase in age. For instance, the control specimen showed an expansion of 0.28%, 0.34%, and 0.37% at 28 days, 56 days, and 150 days, respectively.

For the specimen incorporating various dosages of MS as a replacement of reactive aggregates, the expansion was observed to reduce. For instance, the expansion exhibited by specimens containing 5% MS at 14 and 28 days was 0.21% and 0.28%. Although these values are still larger than the limits prescribed by ASTM C1260, these are lower than the control specimen. This indicatestoincorporatemore MS content, the expansion may be reduced further. The specimen incorporating 10% MS showed an expansion of 0.18% and 0.25% at 14 days and 28 days respectively. A similar trend of reduced expansion was observed with an increase in the MS content. At 20% MS content, the 14 days expansion was found to be 0.13% which was still higher than 0.1%. However, the 28 days expansion was found to be 0.18% which was lesser than the 0.2% limit. Further, at 25% MS incorporation, the 14 days expansion was found to be 0.09% while the 28 days expansion was found to be 0.17%. Both the expansion values were within the ASTM limits. It is evident that a high percentage of MS may be considered viable in reducing ASR expansion. The reason for the reduced ASR expansion by increasing the proportion of MS is the reduced porosity and the reduced overall silica dissolution. The reduction in silica content played an important role in reducing the expansion after replacing aggregate with MS. Similar findings have been reported by other researchers which show a reduction in expansion ofthe incorporation of supplementary materials in concrete mixtures. For instance, Abbas et al. reported a reduction of ASR expansion in mortar specimens on incorporating Marble Power [67]. Afshinnia et al. also noticed a decrease in ASR expansion of about 35% by replacing 10% cement with waste clay brick powder [68]. A similar reduction in expansion was reported elsewhere [48]. A 30% reduction in ASR expansion was reported in a mixture containing 30% sugar cane bagasse ash as compared to the control samples reported by Abbas et al. [3].

### 4.4. Impact of ASR on Mechanical Performance

**Figure 7:**
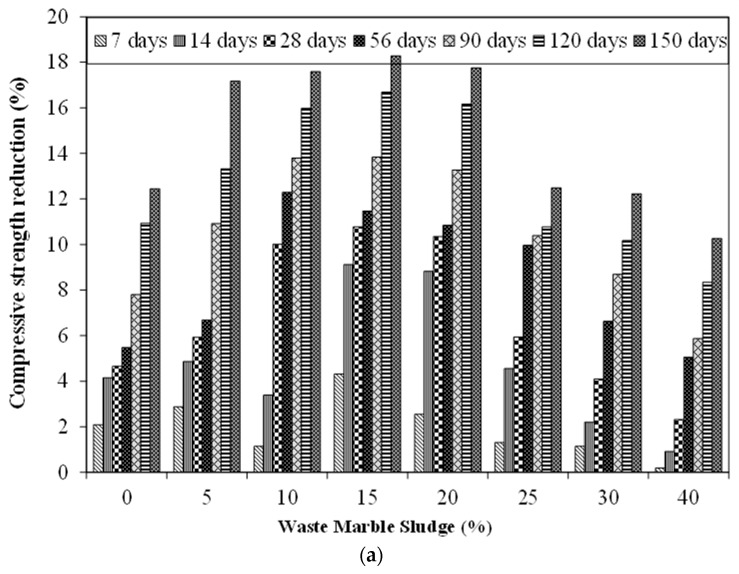

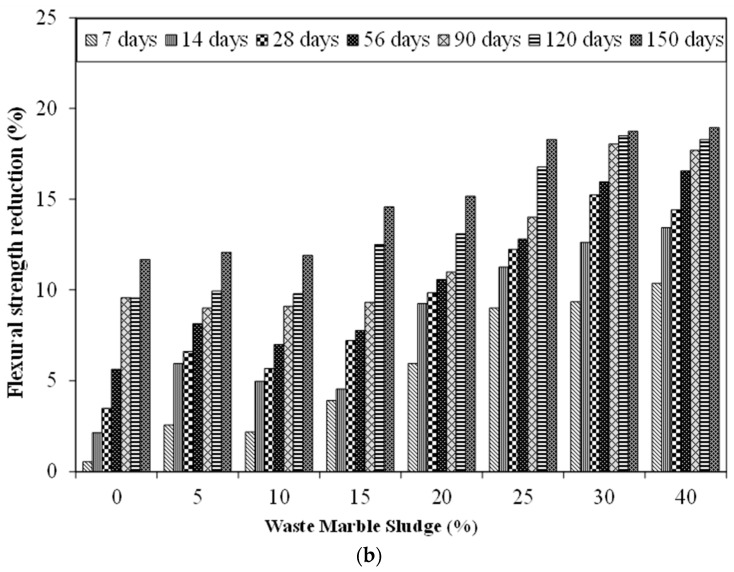
Effect of ASR on MS mortar specimens (**a**) compressive strength reduction due to ASR (**b**) (**a**) flexural strength reduction due to ASR.

Figure 7 show the results pertaining to the reduction in the compressive and flexural strength of specimen prone to ASR environment. Figure 7a shows a reduction in compressive strength of the control specimen of 4.6%, 5.5%, and 12.5% at 28 days, 56 days, and 150 days, respectively, as compared to the compressive strength of identical specimens kept in normal water for curing. This reduction in compressive strength may be attributed to the formation of ASR gel which further causes micro-cracking leading to a reduction in mechanical properties [69,70]. On incorporating MS, the reduction in compressive due to ASR increased slightly. For instance, at 5% MS content the reduction in compressive strength was found to be 6.1%, 6.7%, and 17.1% at 28 days, 56 days, and 150 days, respectively. Similarly, at 20% MS content, the reduction in compressive strength was found to be 10.8%, 10.8%, and 17.8% at 28 days, 56 days, and 150 days, respectively. A similar reduction in compressive strength was reported by earlier studies as well [71]. Similar to compressive strength, a reduction in flexural strength was observed in a specimen subjected to anASR-prone environment. The flexural strength of the control specimen was reduced by 3.8%, 5.6%, and 11.7% at 28, 56, and 150 days respectively. Similarly, the flexural strength of specimen incorporating 20% MS showed a reduction of 11.6%, 10.6%, and 15.2% at 28, 56, and 150 days respectively.

### 4.5. Thermal Analysis

**Figure 8:**
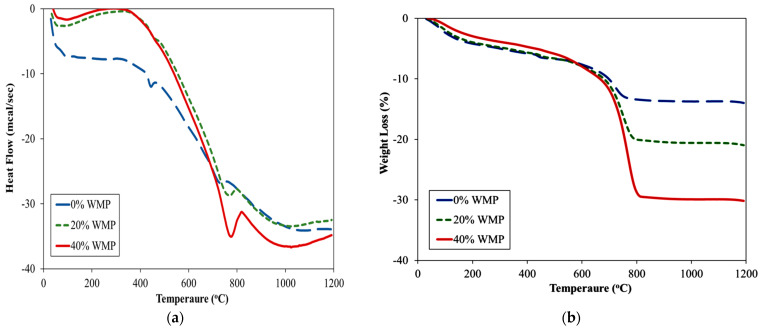
Thermal analysis results of Samples incorporating various dosages of MS (**a**) heatflow and (**b**) weight loss.

Figure 8 shows the results of thermogravimetric analysis (TGA) and differential thermal analysis (DTA) for control specimens and specimens incorporating 20% and 40% MS.

For simplicity, the TGA curves of the samples having different proportions of the MS have been divided into four temperatures ranges: 25–123 °C, 123–430 °C, 480–750 °C, and 750–up to 1200 °C. The mass loss observed within the 25–123 °C range was 3.5% for the control specimen, while it was 2.6% and 1.64% for specimens incorporating 20% and 40% MS, respectively. The mass loss may be attributed to the loss of trapped moisture content in the specimens [72]. The results show greater weight loss in the case of the specimen incorporating MS as compared to the control specimen. Within 123–480 °C range, 3.1%, 3.8% and 4.1% mass loss were observed in specimen incorporating 0%, 20% and 40% MS. This mass loss may be attributed to the CSH. The mass loss observed at 480–730 °C was observed as 9.49%, 9.53%, and 13% having 0%, 20%, and 40% MS, and may be attributed to the decomposition of Ca(OH)_2_. Similarly, the mass loss within 730–1200 °C was 2.1%, 4.6% and 11.9% for specimen incorporating 0%, 20% and 40% MS. This mass loss may be attributed to the disintegration of calcium carbonate CaCO_3_. As at this zone, mass loss increases with the increasing percentage of MS which confirms the calcite form of marble powder [73].

### 4.6. Micro-Structural Analysis

**Figure 9:**
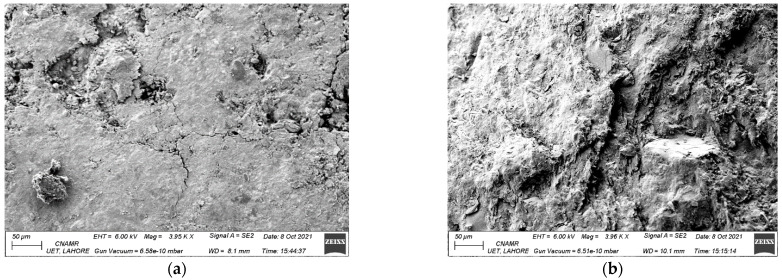

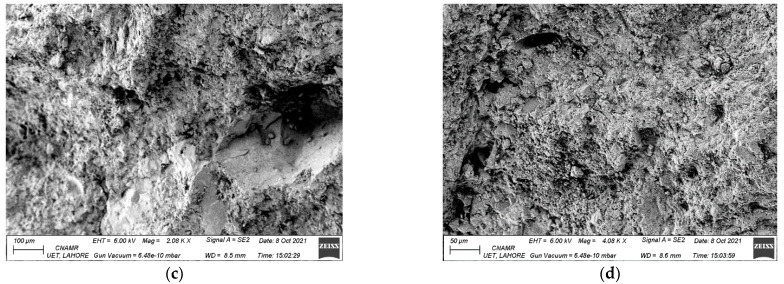
SEM images of specimens with varying percentages of MS (**a**) control specimen (50 μm) (**b**) 10% MS (50 μm) (**c**) 40% MS (100 μm) (**d**) 40% MS (50 μm).

Figure 9 shows the Scanning Electron Microscopy images of the control specimen as well as the specimen incorporating 10% and 40% of MS at 28 days of curing age (curing carried out immersion of the samples in 1N NaOH solution). Figure 9a shows the presence of micro-cracks in the control specimen at 28 days. These microcracks are mainly caused by the expansion of the ASR gel. Previous researchers have observed similar findings. Abbas etal. reported the formation of micro-cracks in the mixtures due to the formation of ASR gel around the aggregate particles [47]. Figure 9b–d shows that on incorporating MS in the mixture, no cracks were developed. The formation of ASR gel was reduced due to a lesser quantity of alkalis available. The findings are synonymous with the previous reports, wherein it was reported that withthe incorporation of supplementary materials composed of lime, silica alumina, etc., the cracking due to ASR is reduced [67,74].

## 5. Conclusions

The utilization of waste marble sludge powder (MS) for mitigating ASR expansion was explored. Various proportions of MS (ranging from 5% to 40%) were used as a replacement for aggregates. The effect of incorporation of MS was observed on the compressive strength, flexural strength, and expansion due to ASR in mortar bar specimens. Based on the experimental results, the following conclusionshave been drawn:

- The incorporation of MS at various dosages exhibits a plateau effect. The compressive strength of the specimen increases with the increased replacement of aggregate with MS. However, this trend continues up to 25% MS content. As per the results, 28 days’ compressive strength was observed to be 29 MPa, 42 MPa, and 26 MPa for specimens incorporating 0%, 20%, and 40% MS, respectively. Maximum strength was observed on the incorporation of 20% MS which was 46% more than the control specimen. At 150 days of curing age, the compressive strength was observed to be 42 MPa, 53 MPa, and 30 MPa for specimens incorporating 0%, 20%, and 40% MS, respectively.
- The incorporation of MS caused an increase in the flexural strength up to 15% MS content. On increasing the MS content beyond 15%, the flexural strength started reducing. At 28 days, the flexural strength of specimen incorporating 0%, 15%, and 40% MS was observed to be 10 MPa, 11 MPa, and 10.6 MPa, respectively. Similarly, at 150 days of age, the flexural strength of specimen incorporating 0%, 15%, and 40% MS was observed to be 12.7 MPa, 14.0 MPa, and 12.7 MPa, respectively.
- The control specimen exhibited expansion of 0.23% and 0.28% at 14 days and 28 days, respectively, when subjected to ASR curing regime as prescribed in ASTM C1260. It was observed that on incorporating MS, the expansion was reduced. The expansion observed in the case of specimens incorporating 20% MS was 0.13% and 0.18% at 14 days and 20 days respectively, where the latter was found within the ASTM C1260 limits. Moreover, the specimen incorporating 25% MS exhibited 0.09% and 0.17% expansion at 14 days and 28 days, respectively. Both these values were within the limits prescribed by ASTM C120. The reduction in expansion was primarily attributed to the reduced alkali content and consequently reduced content of reactive aggregates.
- A reduction in compressive and flexural strength was observed due to ASR conditions. The compressive strength of the control specimen (without MS) was observed as 4.6% and 12.5% at 28 days and 150 days, respectively. Similarly, the reduction in compressive strength in specimens incorporating 20% MS was observed at 10.8% and 17.8% at 28 days and 150 days respectively. A similar reduction in flexural strength was also observed.
- The severe micro-cracks were observed in the case of the control specimen subjected to ASR conditions. The formation of ASR gel was also observed. However, the specimen incorporating MS did not exhibit any signs of micro-cracking, although the formation of relatively lesser ASR gel was observed during microstructural analysis.

Therefore, the utilization of MS can be considered a viable option for mitigating ASR while providing an improvement in the mechanical properties of concrete/cement mortar. As an added value, the dumping issue for this otherwise waste material may also be resolved along with reduced harmful emissions.

## Figures and Tables

**Table 1:** Various proportions of MS used for preparing specimens.

Serial No.	Designation	Percentage Replacement by Aggregate
1	Control	-
2	MS5	5
3	MS10	10
4	MS15	15
5	MS20	20
6	MS25	25
7	MS30	30
8	MS40	40

MS: Marble Sludge; mixture proportion (cement:aggregate = 1:2.25).

**Table 2:** Composition of cement and MS (chemical analysis).

Constituents (%)	Cement	ASTM Limits (Cement)	MS
SiO_2_	20.85	17–25	1.03
Al_2_O_3_	2.49	3–8	0.93
Fe_2_O_3_	3.21	0.5–6.0	0.07
CaO	62.08	60–67	54.4
MgO	1.96	0.5–4.0	0.55
SO_3_	3.48	2.0–3.5	-
Na_2_O	0.10	-	0.07
K_2_O	0.74	-	0.10
Na_2_O_e_	0.58	<0.6	0.13
LOI	2.78	<3.0	42.98
IR	0.43	<0.75	-
C_3_S	71.58	42–67	-
C_2_S	2.23	8–31	-
C_3_A	8.4	5–14	-
C_4_AF	9.66	6–12	-
LSF	1.00	0.66–1.02	-
SR	2.27	2.0–2.5	-
AR	1.64	1.5–2.5	-

**Table 3:** Physical properties of raw materials (cement and MS).

Properties	Cement	ASTM Limits (Cement)	MS	Standards
Specific gravity	3.15	3.10–3.25	2.64	ASTM C188
Unit weight (kg/m^3^)	1400	830–1650	1206	ASTM C29
Fineness (Passing 200 sieve) (%)	90	>90	100	ASTM C204
Blaine fineness (cm^2^/g)	2200	2250	2237	ASTM C184
Autoclave expansion (%)	0.13	0.20	-	ASTM C151
Standard consistency (%)	24.6	-	-	ASTM C187
Initial setting time (Min)	120	>45	-	ASTM C191
Final setting time (Min)	230	<375	-	ASTM C191

**Table 4:** Chemical and physical properties of aggregates used in mixtures.

Tests/Description	Results (%)
**Chemical Properties**	
Water-soluble chloride content (BS 812-117)	0.043
Sulfate content (BS 812-118)	0.014
Soundness by weighted average loss (Na_2_SO_4_), (ASTM C88)	4.658
Soundness by weighted average loss (MgSO4), (ASTM C88)	5.270
Calcium oxide (CaO)	9.890
Magnesium oxide (MgO)	10.320
Loss on ignition (LOI)	15.400
Silica (SiO_2_)	56.920
Alkali metals	0.379
Ferric oxide (Fe_2_O_3_)	1.767
Chromium oxide	0.038
Alumina (Al_2_O_3_)	5.233
**Physical Properties**	
Bulk Density (kg/m^3^) (ASTM C29)	1307.42
Specific Gravity (ASTM C188)	2.6
Water Absorption (%)	2.2
Impact Value (%) (BS 812)	22.31%
Abrasion Test (%) (ASTM C535)	22.30%

## Data Availability

Not applicable.
